# Nitrogen-doped carbons derived from cotton pulp for improved supercapacitors†

**DOI:** 10.1039/d2ra02850f

**Published:** 2022-10-13

**Authors:** Jian Shen, Jiangbin Yu, Hao Luo, Xiang Liu, Qiongzhi Zhou, Tianxiang Wei, Xinyi Yu, Yahui Wu, Yifei Yu, Mingjie Li

**Affiliations:** College of Environment and Resources, Xiangtan University Xiangtan 411105 China js_xtuhzy@xtu.edu.cn; National Key Laboratory of Human Factors Engineering, Chinese Astronaut Research and Training Center Beijing 100094 China lishun2009@163.com; Group of Biomimetic Smart Materials, CAS Key Laboratory of Bio-based Materials, Qingdao Institute of Bioenergy and Bioprocess Technology, Chinese Academy of Sciences Songling Road 189 Qingdao 266101 China limj@qibebt.ac.cn

## Abstract

Supercapacitors have a rapid charge/discharge rate, long lifespan, high stability, and relatively acceptable cost, showing great potential in energy storage and conversion applications. However, the current cost-effective carbon-based electrodes have limited application owing to their low specific capacitance and unsatisfactory stability. In this regard, we herein prepare nitrogen-doped carbons by carbonizing a mixture of cotton pulp (CCP) and melamine to improve the specific capacitance by integrating pore (mesopore) and surface (oxygen-containing groups) modification with defect engineering *via* the carbonization process. Furthermore, the structural and morphological features of the resultant nitrogen-doped carbons are confirmed by various characterization techniques. Excitingly, the specific capacitance for nitrogen-doped CCP (CCPN1) with a 1 : 1 weight ratio of CCP and melamine is 642 F g^−1^ at a current density of 0.5 A g^−1^ in a three-electrode system, surpassing that of the reported carbon analogues and most metal-based materials to date. The stability test suggests that the specific capacitance of CCPN1 is maintained over 150 F g^−1^ at a current density of 2 A g^−1^ even over 5000 cycles. Therefore, the reported nitrogen-doped carbons from cotton pulp exhibit improved specific capacitance and stability, providing a new cost-effective carbon-based material for application in the energy storage field.

## Abstract 2

Supercapacitors have a rapid charge/discharge rate, long lifespan, high stability, and relatively acceptable cost, showing great potential in energy storage and conversion applications.

## Graphical abstract

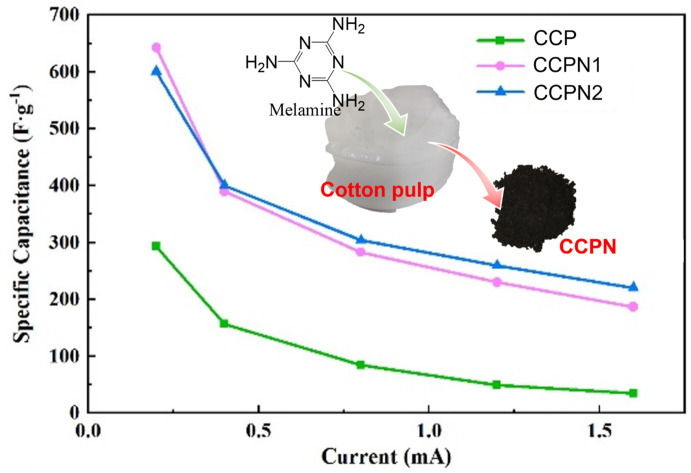

## 1. Introduction

With the continuous over-consumption of fossil fuels and the exacerbation of global climate issues, high-efficiency electricity storage technologies have attracted great attention due to their carbon-neutral and environmentally benign nature.^1^ Among the current strategies, supercapacitors possess rapid charge/discharge rates, long lifespans with high stability, and relatively acceptable cost for either individual usage or supplemented with batteries in the fields of energy storage and conversion,^2^ where their performance in electrical double-layer capacitors (EDLCs) and pseudocapacitors mainly rely on the electrostatic adsorption/desorption of ions and local redox pairs over the surface of electrodes, respectively.^3^ Although tremendous efforts have been made in the development of materials to improve the performance of supercapacitors, carbon-based materials are still considered ideal electrode material candidates for supercapacitors due to their flexible and tunable porous structures,^4^ surface functional groups,^5^ and foreign atoms,^6^ which have enormous impact on the capacitance of EDLCs and pseudocapacitors. For instance, Wang *et al.* reported that the pore size distribution, rather than the specific surface area, has a tremendous impact on ion adsorption/desorption over the electrode surface. Specifically, ion clusters always cross over the micropores in irreversible capillary condensation, which blocks the pathways of ion adsorption/desorption, thereby compromising EDLC performance.^7^ For carbons with a hierarchically porous structure originating from a source of wood, macro–mesopore/meso–micropore compounds show an optimal electrochemical performance of 133 F g^−1^ (27 F cm^−3^) at 10 mA cm^−2^, outperforming currently reported wood biochar monoliths. Surface functional groups, especially oxygen-containing groups, strengthen the electrochemical performance due to their positive charge and subsequently promote electron transfer during the charging/discharging process.^8^ Doping foreign atoms,^9,10^ such as N and S, also provide numerous local redox pairs to boost the pseudocapacitance by redistributing electrons over the active sites. Therefore, synergistically realizing hierarchically porous carbons with oxygen-based groups and heteroatoms is deemed a desirable channel for boosting the electrochemical performance of supercapacitors.

Previously, most carbon-based electrodes for supercapacitors have been mainly produced using fossil fuel-based precursors (coal, polymers, pitch and so forth) *via* thermal carbonation, resulting in enormous nonrenewable energy resource consumption and leaving large carbon footprints.^11,12^ However, recent research has reported that wood-derived biochar shows huge potential in the energy storage field due to the following aspects: (1) as a renewable and sustainable resource, the cost-effective nature and high-performance of wood can replace non-renewable, fossil fuel-based materials in the environment–energy nexus;^13,14^ (2) anisotropic pores with hierarchical cellular structures provide channels for water and ion transfer and regulate the corresponding rate, enhancing the performance of EDLCs; (3) the large number of oxygen-containing groups and heteroatoms afford local redox sites, potentially promoting pseudocapacitance. Thus, based on these merits of wood precursors, materials using bamboo,^15^ poplar,^16,17^*Prosopis juliflora* wood,^18^ beech,^19^ and cotton pulp^20^ have been reported. Of the reported wood-based carbons, cotton pulp has attracted tremendous attention due to its homogenous pore size, stable hierarchical porous architecture^21^ even when carbonized at high temperature, and cost-effective and environmentally friendly nature.^20^ Jiang *et al.* adopted a two-step carbonization process to convert cotton pulp into carbons, which exhibited a characteristic capacitance of 107 F g^−1^ at 1 A g^−1^ with acceptable stability for 2000 cycles,^20^ which crossed our expectations. Therefore, based on the previous discussion, the aim of this work is to utilize the advantages of cotton pulp and synergistically combine pore (mesopore) and surface (oxygen-containing groups) engineering with defect engineering (N dopant) to boost the performance of EDLCs and pseudocapacitors *via* a one-step carbonization process of a cotton pulp and melamine mixture to achieve superior capacitance with exceptional stability. Furthermore, diverse structural characterizations, including thermogravimetric analysis (TGA), Brunner–Emmett–Teller (BET) analysis, Raman spectroscopy, and X-ray photoelectron spectroscopy (XPS), have been conducted from the perspectives of structural, defective, and surface functional group features. Finally, the performance of supercapacitors decorated with nitrogen-doped carbon has also been evaluated.

## 2. Experimental

### 2.1 Chemicals

All the chemicals, including cotton pulp (diameter: 5–20 nm, length: 10–1000 nm, purity: 99.9%, crystallinity ≥ 75%, solid content ≥ 5%, North Century (Jiangsu) Cellulose Materials Co., Ltd.), melamine (AR 99%, Aladdin), Vulcan XC-72 carbon (Cabot), and Nafion (5 wt%, DuPont D520) were used as received without any purification.

### 2.2 Preparation of nitrogen-doped carbon derived from cotton pulp

First, the purchased cotton pulp was dehydrated by vacuum drying to protect the textural structure of cellulose nanofibers for forty-eight hours after being firmly frozen at −70 °C for twelve hours. Afterwards, mixtures of different weight ratios (1 : 0, 1 : 1, and 2 : 1, labeled as CCP, CCPN1, and CCPN2, respectively) of melamine and dried CCP were ground at room temperature for at least thirty minutes until a homogenous mixture was formed. Then, the mixture was annealed under an argon atmosphere in a tube furnace with a temperature ramp of 10 °C min^−1^ from room temperature to 550 °C with a one-hour platform, followed by further increasing the temperature to 800 °C with the same rate and keeping it at 800 °C for another two hours. Finally, the prepared samples were obtained and stored in a drying cabinet for further testing.

### 2.3 Preparation of electrodes

2 mg of the synthesized materials (CCP, CCPN1, and CCPN2), 0.25 mg carbon black, and 5 mg Nafion (5 wt%) were dispersed in a 275 μL mixed solution of isopropanol and DI water with a 3 : 7 volume ratio. Then, the suspension was vortexed and sonicated for at least 10 minutes three times to ensure the formation of homogenous ink. Afterwards, the ink was drop-casted on hydrophobic carbon paper with a size of 1.0 cm × 1.0 cm and dried at room temperature until the solvent completely evaporated, and a loading of 0.4 mg cm^−2^ was maintained for all the electrodes.

### 2.4 Electrochemical measurements

The nitrogen-doped porous wood-derived carbons (CCP, CCPN1, and CCPN2) were used as prepared. An electrochemical workstation (IVIUM, V22612) was used as the voltage/potential/current source for three-electrode electrochemical analyses. In the three-electrode setup, the catalyst-coated carbon paper was connected to the working electrode terminal. The counter and reference electrode terminals were Pt slice and Ag/AgCl electrodes, respectively. The electrochemical performance of the supercapacitors was tested by cyclic voltammetry (CV) and galvanostatic charge/discharge (GCD) in 0.1 M NaCl. The potential range for CV and GCD was from −1.0 to +1.0 V. The mass-specific capacitances (*C*) were calculated using the equation *C* = 4(*I*Δ*t*)/(*m*Δ*V*), where *I* is the constant discharging current, Δ*t* is the discharging time, *m* is the mass of the electrode materials, and Δ*V* is the potential drop during the discharge process in the range of *V*_max_ and 1/2*V*_max_.^20^

### 2.5 Characterization

Thermogravimetric analysis (TGA) was conducted on an STA449 F5 (Netzsch, Germany) with a ramp of 10 K min^−1^ from room temperature to 800 °C by inletting N_2_ as the carrier gas. The microstructures of the samples were observed by scanning electron microscopy (SEM, Hitachi S-4800). The textural properties of the samples were identified by nitrogen adsorption/desorption isotherm analysis examined at 77 K on a NOVA-2200e analyzer (Quantachrome, USA). The defects and graphitization of the samples were analyzed by recording their Raman spectra (InVia, Renishaw, PLC, UK). The surface functional groups were analyzed by X-ray photoelectron spectroscopy (XPS) (ESCALAB 250 XI spectrometer).

## 3. Results and discussion

### 3.1 Structural and morphological characterization

**Fig. 1:**
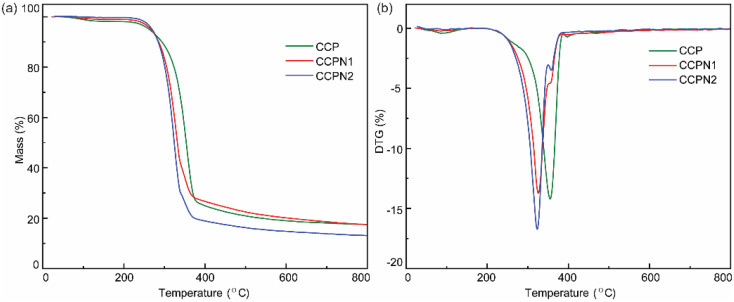
(a) Thermogravimetric analysis (TGA) and (b) derivative thermogravimetry (DTG) curves of CCP, CCPN1, and CCPN2.

To confirm the formation of N-doped cotton pulp-derived carbons, thermogravimetric analysis (TGA) was performed. As presented in Fig. 1a, the TGA profiles of CCP, CCPN1, and CCPN2 exhibit a relatively similar tendency of mass loss as the temperature increases from room temperature to 800 °C with a rate of 2 °C min^−1^. Specifically, the remarkable mass loss at the temperature range of 200 °C to 400 °C can be attributed to the thermal decomposition of cellulose,^22,23^ a component of the cotton pulp.^24^ However, in comparison to pure CCP, the slight differences in the TGA curves of CCPN1 and CCPN2 suggest that the inflection point of mass loss at 300–400 °C is mainly ascribed to the further transformation from melamine to C_3_N_4_ (Fig. 1a).^22^ The continuous increase in temperature gives rise to C_3_N_4_ decomposition, resulting in the second platform of mass loss until the temperature reaches 800 °C.^25^ For further investigation, DTG curves were plotted to probe the nitrogen doping behavior. The results in Fig. 1b suggest that, unlike for pure CCP, the characteristic peak in the vicinity of 330 °C in the DTG curve of the CCP and melamine mixture indicates the occurrence of a cross-linking reaction between melamine and the hydroxyl groups on the surface of CCP (O 1s XPS spectrum in Fig. S1†), which leads to the formation of nitrogen-doped CCP, as shown in Fig. 3a. Additionally, such a peak indicates that the mesopore protect CCP by limiting the shrinkage of CCP during the carbonization process, which agrees with the data of its textural properties (Table S1†).

**Fig. 2:**
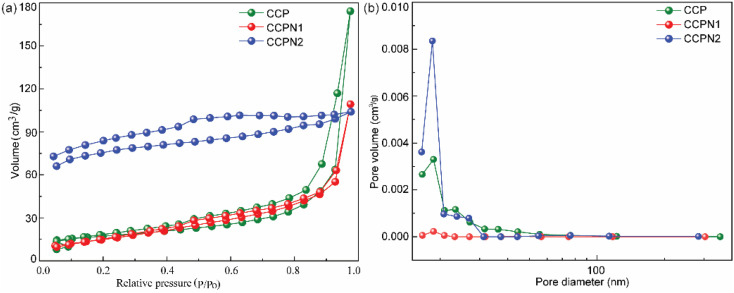
(a) Nitrogen adsorption/desorption isotherms and (b) pore size distribution curves of CCP, CCPN1, and CCPN2.

The morphology of the mentioned materials was characterized by scanning electron microscopy (SEM). As shown in Fig. S2,† pure CCP exhibits a lump structure with a partially wrinkled surface. As the doping amount of nitrogen increases, the surface of the CCPN series of carbons becomes rough, probably resulting from the thermal decomposition of melamine. To further identify the textural properties (specific surface area and pore size distribution), the nitrogen adsorption/desorption isotherms are recorded. As shown in Fig. 2a, all the samples show type IV isotherms as classified by the IUPAC. Specifically, the hysteresis loop occurs over the entire investigated pressure range, without presenting steep nitrogen uptake at low partial pressure (*P*/*P*_0_ < 0.1). Therefore, it is suggested that unique mesopores dominate the main porous structure.^26,27^ Additional information from pore size distribution (PSD) (Fig. 2b) showcases that the central peaks in the PSD of CCP, CCPN1, and CCPN2 are at 18.66 nm, 18.66 nm, and 18.45 nm, respectively, providing supplementary proof of the presence of mesopores (size: ∼18.5 nm, summarized in Table S1†) and suggesting the stability of the mesoporous structure after nitrogen doping, which grants numerous active sites for ion adsorption and quick ion migration.^28^ Excitingly, it is worth noting that the specific surface area (351.8 m^2^ g^−1^) of CCPN1 is much higher than that of CCP (30.6 m^2^ g^−1^) and CCPN2 (252.6 m^2^ g^−1^), potentially providing plenty of adsorption sites.

**Fig. 3:**
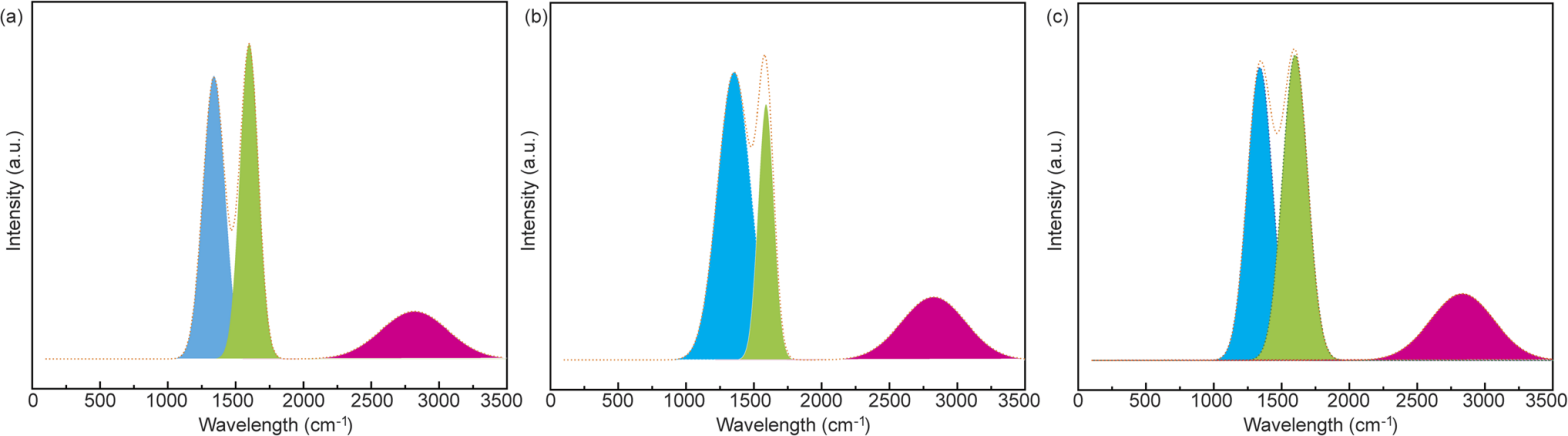
Raman spectra of (a) CCP, (b) CCPN1, and (c) CCPN2.

To identify the defects or graphitization of carbon-based materials, Raman spectroscopy is employed by focusing on two characteristic peaks at ∼1350 cm^−1^ (D band) and ∼1580 cm^−1^ (G band).^29^ Specifically, a higher area ratio of the D and G bands (*I*_D_/*I*_G_) indicates more defects and lower graphitization levels. As shown in Fig. 3, the *I*_D_/*I*_G_ values of CCP, CCPN1, and CCPN2 are 0.902, 0.995, and 0.993, respectively, indicating lower graphitization induced by more defects from melamine decomposition. However, in porous materials, judgments based on the value of *I*_D_/*I*_G_ can offer misleading information on the graphitization crystal structure as porous channels will impart the graphitization level of only the bulk counterpart, neglecting the local graphitic or ordering domains.^27^ Therefore, it is better to obtain the graphitic ordering structure for porous carbons by investigating the narrowness of the G and D bands.^29^ As clearly observed in Fig. 3, the value of the D band of CCPN1 is much wider than that of CCP (165–180 cm^−1^), suggesting a lower level of graphitization or ordering in CCPN1. Moreover, the higher ratio of D and G band narrowness serves as complementary proof to confirm the defect structure in CCPN1. In addition, the 2D′ Raman peak centered at ∼2700 cm^−1^ is highly correlated with the disorder level of heteroatom-doped graphite. Specifically, a higher intensity indicates a more disordered structure in carbon-based materials after doping heteroatoms.^30^ As seen in Fig. 3, the intensity of the 2D′ Raman peak is promoted as more nitrogen is doped into the carbons, indicating a more disordered structure in nitrogen-doped CCP, which is consistent with the conclusion of the discussion of the D and G bands.

**Fig. 4:**
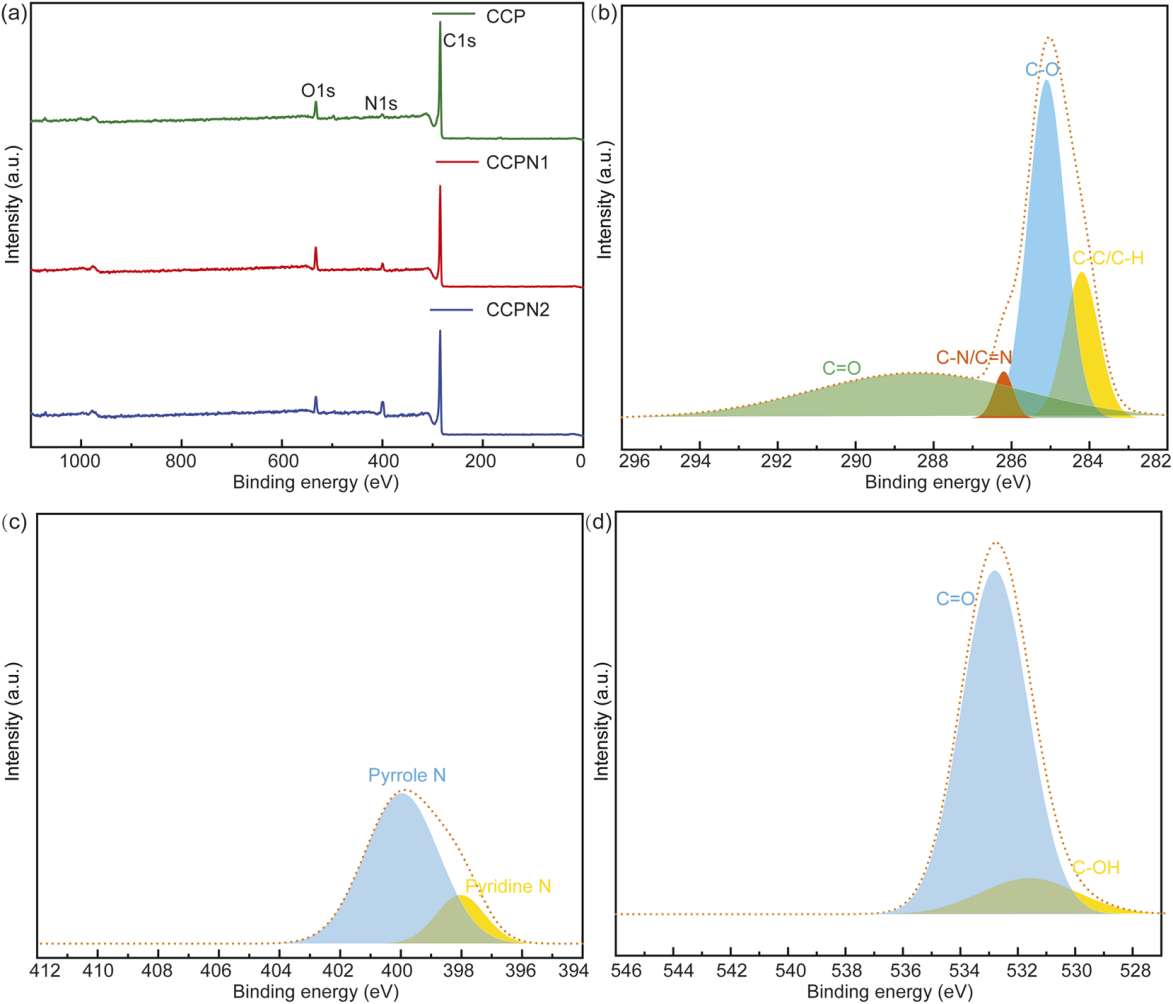
(a) Survey XPS spectra of CCP, CCPN1, and CCPN2. (b–d) XPS spectra of C 1s, N 1s, and O 1s in CCPN1, respectively.

For further investigation on the impact of the surface functional groups of CCP and nitrogen-doped CCP, the survey XPS spectra and XPS spectra of the C, N, and O elements in CCPN1 are shown in Fig. 4. As controls, the XPS spectra of CCP and CCPN2 are presented in Fig. S1 and S3–S7.† According to the data from Fig. 4a, it is clearly seen that the intensity of the N signal is enhanced as the amount of nitrogen doping increases. Meanwhile, the intensity of the O signal presents a “volcano” type, indicating that the highest intensity is achieved when the predetermined weight ratio of CCP and melamine is 1 to 1. Specifically, the N content increased from 3.18 to 11.68 atom%. Notably, the N content of pure CCP probably comes from cotton pulp, consistent with previous observations that multiple non-carbon inorganic atoms exist in cotton pulp.^17^ For the O element, the maximum content is achieved at 10.01 atom% by decomposing the mixture of CCP and melamine with a 1 : 1 weight ratio, outperforming the corresponding value of either pure CCP (6.44 atom% in CCP) or a higher melamine weight ratio (6.83 atom% in CCPN2) (Table S2†). Furthermore, as shown in Fig. 4b–d, the main surface functional groups include pyrrole-N/pyridine-N^31^ and C=O/C–OH, which promote the local redox environment and ion adsorptive sites, synergistically boosting the performance of pseudocapacitors and EDLCs, respectively.

### 3.2 Electrochemical performance

**Fig. 5:**
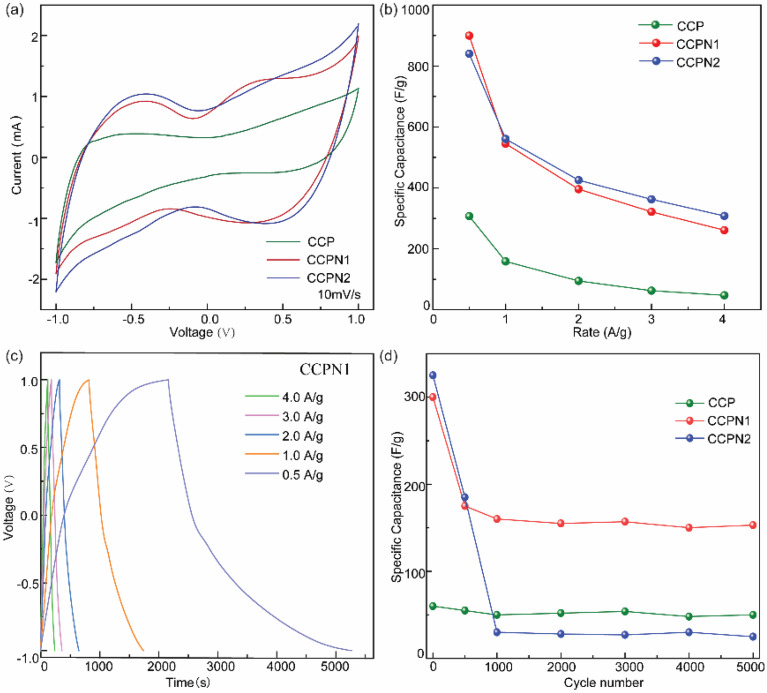
(a) Cyclic voltammograms in 0.1 M NaCl scanned from 1.0 to −1.0 V. (b) The correlation between specific capacitance and current. (c) GCD curves in 0.1 M NaCl of CCPN1 at different current densities. (d) Specific capacitance of the investigated carbon-based materials against cycle number.

To evaluate the performance of the electrochemical capacitors, cyclic voltammetry (CV) and galvanostatic charge–discharge (GCD) cycling were utilized. Fig. S8–S10† present the CV curves of CCP and N-doped CCP obtained at different scan rates with a potential windows from −1.0 to 1.0 V.^32^ The quasi-rectangular shape of the CV curve with visible redox peaks suggests that capacitive behaviors are still dominated by the EDL, accompanying the contribution of pseudocapacitance. However, as the scan rate increases, the CV curve presents obvious distortion (Fig. S8–S10†), which is probably due to the limited diffusion and migration of electrolytes, agreeing with the observation of Liu *et al.*^17^ Therefore, to determine the effects of nitrogen dopant on the capacitance of the carbonized CCP microporous carbon from the CV curves, we selected a scan rate of 10 mV s^−1^ (Fig. 5a). As observed by Chen *et al.*,^14^ the capacitance mainly relies on the total voltammetric charge from the integration of the positive and negative sweeps between *E*_lowest_ and *E*_highest_ in the CV curve, excluding catalyst mass and scan rate, under the same electrode preparation protocol and test conditions. As shown in Fig. 5a, the integrated area of the CV curve indicates that the capacitance of CCP is enhanced significantly by inducing additional pseudocapacitance *via* the nitrogen heteroatom dopant strategy.

Furthermore, this capacitance variation is also confirmed at different scan rates during the rapid charge–discharge process. As shown in Fig. 5b, the capacitance of nitrogen-doped CCP was much higher than that of individual CCP, suggesting the positive role of redox-induced pseudocapacitance from the nitrogen dopant. Specifically, the specific capacitance of pure CCP is 300 F g^−1^ at 0.5 A g^−1^, approaching that of most carbon materials, as reported in the literature (Table S3†). Excitingly, nitrogen-doped CCP exhibits much higher specific capacitance than pure CCP (Fig. 5b), even surpassing most metal-free materials. Specifically, the specific capacitances for nitrogen-doped CCP with 1 : 1 and 1 : 2 weight ratios of CCP and melamine are 642 and 598 F g^−1^, respectively, at a current density of 0.5 A g^−1^. Even at a higher current density, such as 4.0 A g^−1^, a specific capacitance of 250 F g^−1^ is obtained. Although a higher specific capacitance is achieved at a low current density, the time-consumption impedes its application during the charge–discharge process. Moreover, as the current density increases, the interfacial voltage-drop leads to a decrease in the specific capacitance. For example, the values of the voltage-drop in CCP, CCPN1, and CCPN2 from 0.5 A g^−1^ to 4 A g^−1^ are 0.492 V, 0.223 V, and 0.192 V, respectively, resulting in the reduction in specific capacitance at the corresponding current density range (Fig. 5c). Additionally, insufficient mass transport over the electrode/electrolyte interface at a high current density negatively affects the specific capacitance.^22,27^ Therefore, the long-term cycling stabilities of CCP and nitrogen-doped CCP have been evaluated at a moderate current density of 2 A g^−1^ for 5000 cycles. As displayed in Fig. 5d, pure CCP shows extraordinary stability but only limited specific capacitance. In comparison, although CCPN1 is less stable, the specific capacitance remains greater than 150 F g^−1^ after 5000 cycles. However, on further increasing the nitrogen content, the specific capacitance of CCPN2 shows great collapse as the cycles increase, and is even lower than that of CCP after 1000 cycles.

## 4. Conclusion

Overall, an improved nitrogen-doped biochar from cotton pulp for supercapacitor application has been prepared by the synergistic carbonization of dry cotton pulp and melamine to combine pore and surface modification with defect engineering. The specific capacitance and stability of the nitrogen-doped carbons are highly dependent on nitrogen content, which present a broad size distribution as well as numerous oxygen-terminated functional groups and carbon defects. Excitingly, such a unique structure is beneficial to the performance of pseudocapacitors and EDLCs, affording 642 F g^−1^ at 0.5 A g^−1^ in a three-electrode system, which surpasses the performance of the carbon analogues and most metal-based materials to date. In addition, the specific capacitance of the nitrogen-doped biochar from wood-derived cellulose is maintained at 150 F g^−1^ at a current density of 2 A g^−1^ over 1000 cycles. Based on this performance, the presented biochar can fully utilize waste renewable biomass to prepare advanced carbonaceous energy storage materials.

## Author contributions

Jian Shen, Jiangbin Yu and Hao Luo: conceptualization, methodology, data curation, and writing – original draft; Tianxiang Wei, Xinyi Yu, Yahui Wu and Yifei Yu: investigation, formal analysis and writing; Xiang Liu, Qiongzhi Zhou and Mingjie Li: supervision, review & editing, and conceptualization.

## Conflicts of interest

The authors declare no competing interests.

## Supplementary Material

RA-012-D2RA02850F-s001
