# Supplementary material for: Nitrogen-doped carbons derived from cotton pulp for improved supercapacitors
Source: RSC Adv. 2022 Oct 13;12(45):29246–52. doi: 10.1039/d2ra02850f (PMC9557320; doi:10.1039/d2ra02850f)
Supplement: RA-012-D2RA02850F-s001 [file RA-012-D2RA02850F-s001.pdf]

Electronic Supplementary Material (ESI) for RSC Advances.
This journal is © The Royal Society of Chemistry 2022

Supporting Information

Nitrogen doping carbons derived from cotton pulp for outperformed supercapacitor

Jian Shen[1*], Jiangbin Yu[1], Hao Luo[1], Xiang Liu[2*], Qiongzhi Zhou[1], Tianxiang Wei[1], Xinyi Yu[1], Yahui Wu[1], Yifei Yu[1], Mingjie Li[3*]

[1] *College of Environment and Resources, Xiangtan University, Xiangtan 411105, China*

[2] *National Key Laboratory of Human Factors Engineering, Chinese astronaut research and training center, Beijing 100094, China*

[3] *Group of Biomimetic Smart Materials, CAS Key Laboratory of Bio-based Materials, Qingdao Institute of Bioenergy and Bioprocess Technology, Chinese Academy of Sciences, Songling Road 189, Qingdao 266101, P. R. China*

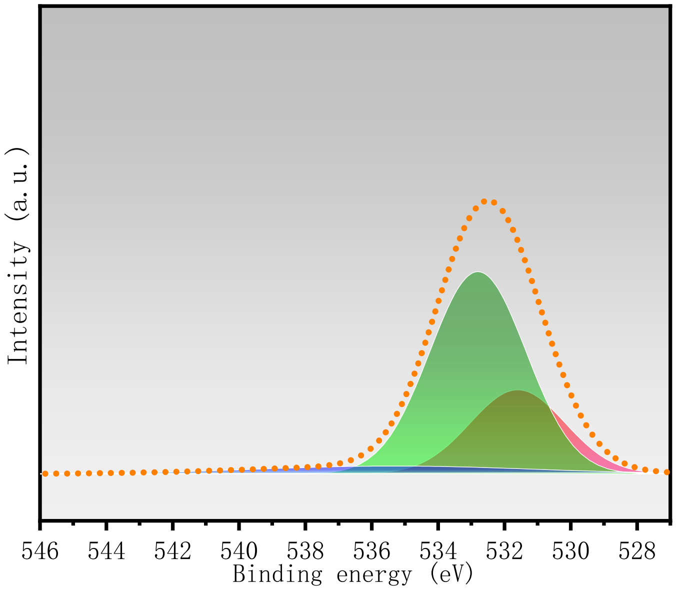

Figure S1 O1s XPS spectrum of CCP

Table S1. Textural properties for the various porous carbon materials utilized in this work

Sample	Specific surface area (m^2/g)	Pore volume (cc/g)	Average pore size (nm)
CCP	30.593	0.030	18.659
CCPN1	351.811	0.032	18.659
CCPN2	252.565	0.038	18.45

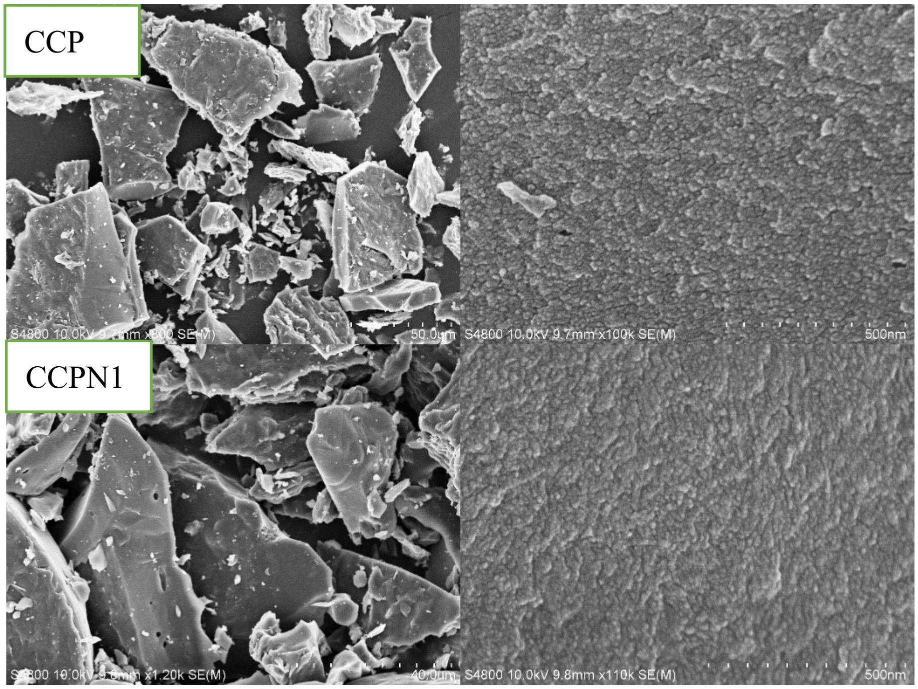

Figure S2. SEM images of pure CCP and nitrogen doping CCP

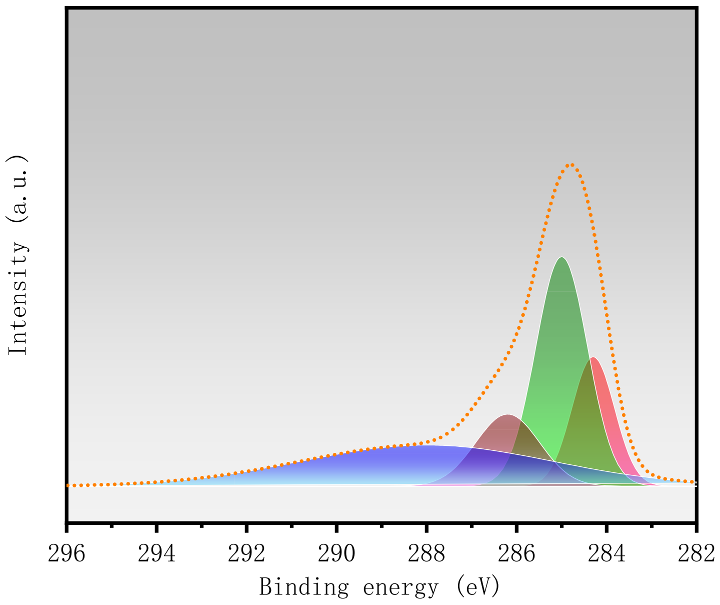

Figure S3. C1s XPS spectrum of CCP

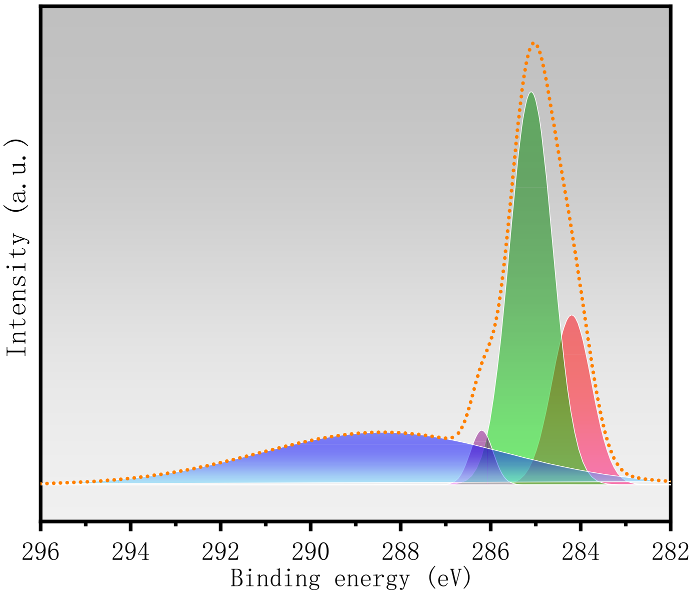

Figure S4. C1s XPS spectrum of CCPN2

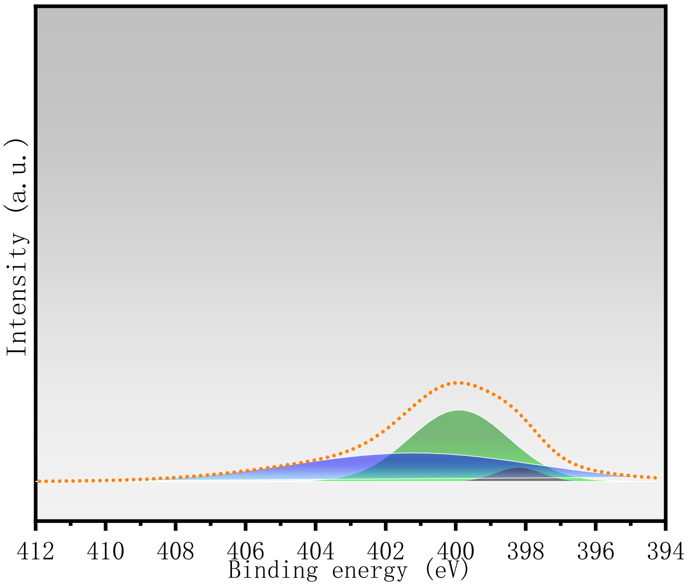

Figure S5. N1s XPS spectrum of CCP

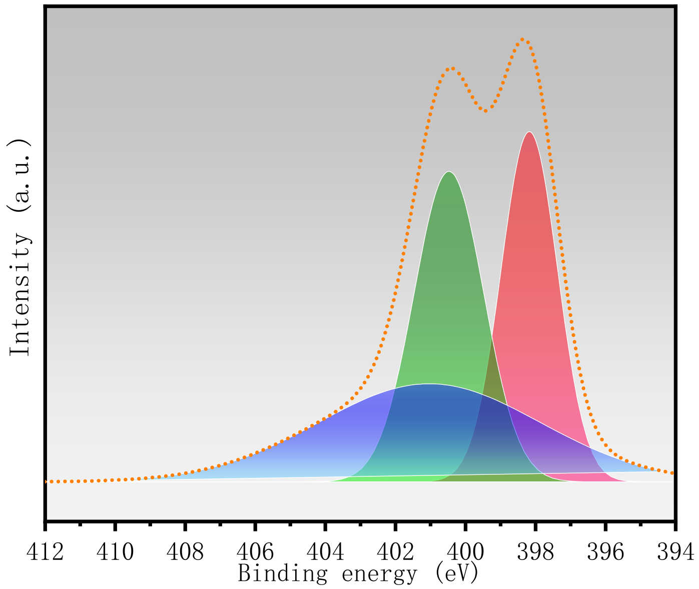

Figure S6. N1s XPS spectrum of CCPN2

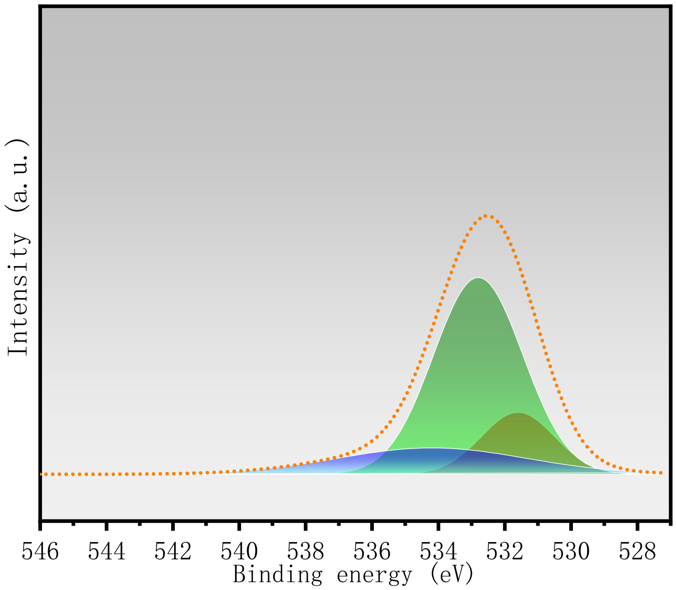

Figure S7. O1s XPS spectrum of CCPN2

Table S2. C, N, and O element atomic ratio in CCP, CCPN1, and CCPN2

Sample	C (Atomic %)	N (Atomic %)	O (Atomic %)
CCP	90.38	3.18	6.44
CCPN1	84.35	5.64	10.01
CCPN2	81.49	11.68	6.83

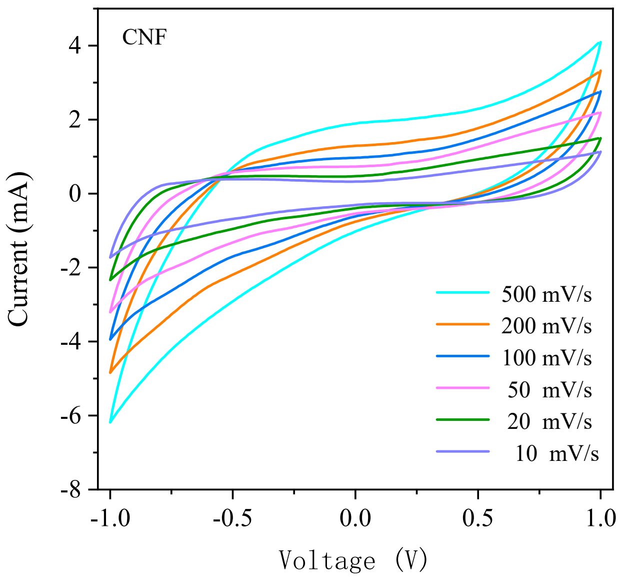

Figure S8. Cyclic voltammograms of CCP in 0.1 M NaCl solution scanning from 1.0 to -1.0V vs Ag/AgCl under different scan rate

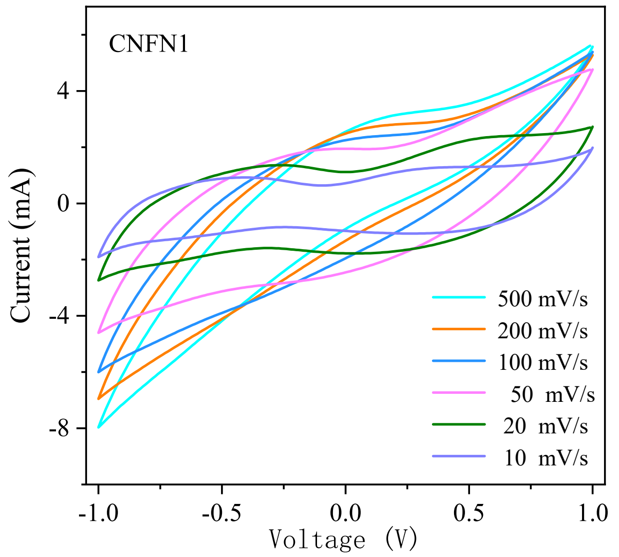

Figure S9. Cyclic voltammograms of CCPN1 in 0.1 M NaCl solution scanning from 1.0 to -1.0V vs Ag/AgCl under different scan rate

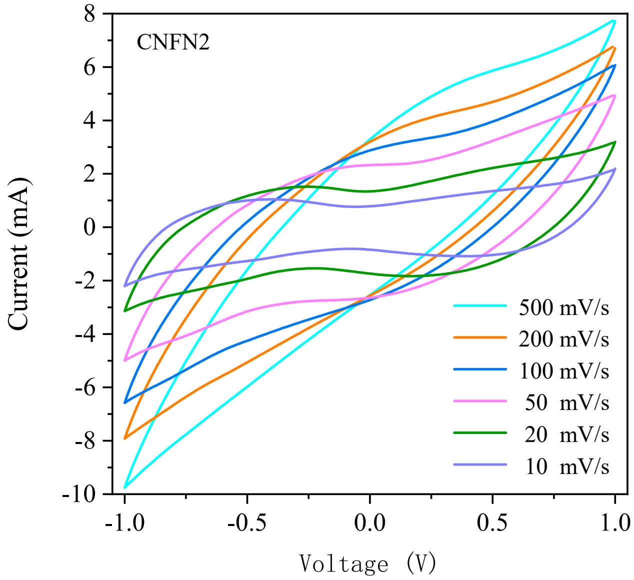

Figure S10. Cyclic voltammograms of CCPN2 in 0.1 M NaCl solution scanning from 1.0 to -1.0V vs Ag/AgCl under different scan rate

Table S3. Comparison of electrochemical performance of CNFN1 with other different materials

Materials	Specific capacitance （F/g）	Current density	Cycling stability	Electrolyte	Ref.
Porous carbon nanofiber	140.80	$0.50 A \cdot g^{-1}$	95.4% capacity retention after 10000 cycles at 10 $A\ g^{-1}$	6 M KOH	1
RuO_2-CNF(220)	188.00	$1.00 mA \cdot cm^{-2}$	93% capacity retention after 3000 cycles at $1.00 mA \cdot cm^{-2}$	6 M KOH	2
CNFs/3DGN	59.28	0.17 $mA \cdot cm^{-2}$	93% capacity retention after 10000 cycles at 0.17 $mA \cdot cm^{-2}$	6 M KOH	3
CNFs	80.00	$0.30 A \cdot g^{-1}$	100% capacity retention after 2000 cycles at 1.5 $A \cdot g^{-1}$	6 M KOH	4
NiCoeBH41@TiC/CNF	2224.00	$0.50 A \cdot g^{-1}$	91% capacity retention after 3000 cycles at 5.0 $A \cdot g^{-1}$	6 M KOH	5
E-CNFs	320.20	$20.00 A \cdot g^{-1}$	94.5 % capacity retention after 5000 cycles at 1.0 $A \cdot g^{-1}$	6 M KOH	6
NiO/C@CNF	742.20	$1.00 A \cdot g^{-1}$	88 % capacity retention after 5000 cycles at 2.0 $A \cdot g^{-1}$	3 M KOH	7
CNF/$MnFe_2O_4$	291.87	$5.00 A \cdot g^{-1}$	Over 80 % capacity retention after 1000 cycles at 5.0 $A \cdot g^{-1}$	2 M KOH	8
Co_2P@N&P-CNFs	475.80	$0.50 A \cdot g^{-1}$	91.5 % capacity retention after 10000 cycles at 0.3 $A \cdot g^{-1}$	1 M KOH	9
N(Ni)-CNF(S)	203.40	$1.00 A \cdot g^{-1}$	94 % capacity retention after 10000 cycles at 1.0 $A \cdot g^{-1}$	1 M KOH	10
Ni-G-CNFs@PANI	318.00	$0.50 A \cdot g^{-1}$	85.8 % capacity retention after 1000 cycles at 10 $A \cdot g^{-1}$	1 M H_2SO_4	11
N(11 wt %)-CNFs	495.00	$0.50 A \cdot g^{-1}$	94 % capacity retention after 10000 cycles at 1.0 $A \cdot g^{-1}$	1 M H_2SO_4	12

PANi/CNF	493.75	1.00 mA·cm^{-2}	94.3 % capacity retention after 5000 cycles at 1.00 mA·cm^{-2}	1 M H_2SO_4	13
3D N-CNFs/V_2O_5	595.10	0.5A·g^{-1}	100 % capacity retention after 12000 cycles at 0.5A·g^{-1}	1 M Na_2SO_4	14
CSS-based graphite / PEDOT / MnO_2	195.70	0.50A·g^{-1}	81 % capacity retention after 2000 cycles at 0.5A·g^{-1}	0.5 M Na_2SO_4	15
Hierarchically porous carbons	383	10 mV·s^{-1}	72% capacity retention after 10000 cycles at 1 A·g^{-1}	1 M KOH	16
**CCPN1**	**642.50**	**0.50A·g^{-1}**	**Over 150 F/g capacity retention after 5000 cycles at 2 A·g^{-1}**	**0.1 M NaCl**	**This work**

References

[1] He, G.; Song, Y.; Chen, S.; Wang, Li. Porous carbon nanofiber mats from electrospun polyacrylonitrile/polymethylmethacrylate composite nanofibers for supercapacitor electrode materials. *J. Mater. Sci.* **2018**, 53, 1–10.

[2] Jeon, S.; Jeong, J. H.; Yoo, H.; Yu, K. H.; Kim, B. H.; Kim, M. H. RuO_2 nanorods on electrospun carbon nanofibers for supercapacitors. *ACS Appl. Nano Mater*. **2020**, 3, 3847–3858

[3] Lin, C. H.; Tsai, C.H.; Tseng, F. G.; Chen, I. C.; Hsieh, C. K. Electrochemical pulse deposition of Ni nanoparticles on the 3D graphene network to synthesize vertical CNFs as the full-carbon hybrid nanoarchitecture for supercapacitors. *Mater. Lett*. **2017**, 192,40–43.

[4] Daraghmeh, A.; Hussain, S.; Servera, L.; Xuriguera, E.; Cornet, A.; Cirera, A. Impact of binder concentration and pressure on performance of symmetric CNFs based supercapacitors. *Electrochim. Acta*. **2017**, 245, 531–538.

[5] Zhou, G.; Xiong, T.; He, S.; Li, Y.; Zhu, Y.; Hou, H. Asymmetric supercapacitor based on flexible TiC/CNF felt supported interwoven nickel-cobalt binary hydroxide nanosheets. *J. Power Sources.* **2016**, 317, 57–64.

[6] Dai, Z.; Ren, P. G.; He, W.; Hou, X.; Ren, F.; Zhang, Q.; Jin, Y. L. Boosting the electrochemical performance of nitrogen-oxygen co-doped carbon nanofibers based supercapacitors through esterification of lignin precursor. *Renew. Energ.* **2020**, 162, 613–623.

[7] Shin, S.; Shin, M. W. Nickel metal–organic framework (Ni-MOF) derived NiO/C@CNF composite

for the application of high performance self-standing supercapacitor electrode. Appl. Surf. Sci. **2020**, 540, 148295.

[8] Sukanya, N.; Pinit, K.; Santi, M. The structural and electrochemical properties of CNF/$MnFe_2O_4$ composite nanostructures for supercapacitors. *Mater. Chem. Phys*. **2018**, 220, 190–200.

[9] Sun, X.; Liu, H.; Xu, G.; Bai, J.; Li, C. Embedding Co_2P nanoparticles into N&P co-doped carbon fibers for hydrogen evolution reaction and supercapacitor. *Int. J. Hydrog. Energy*. **2021**. 46. 1560–1568.

[10] Amiri, A.; Conlee, B.; Tallerine, I.; Kennedy, W. J.; Naraghi, M, A novel path towards synthesis of nitrogen-rich porous carbon nanofibers for high performance supercapacitors. *Chem. Eng. J.* **2020**, 399, 125788.

[11] Tian, D.; Lu, X.; Nie, G.; Gao, M.; Song, N.; Wang, C. Growth of polyaniline thorns on hybrid electrospun CNFs with nickel nanoparticles and graphene nanosheets as binder-free electrodes for high-performance supercapacitors. *Appl. Surf. Sci.* **2018**, 458, 389–396.

[12] Ghanashyam, G.; Jeong, H. K. Synthesis of nitrogen-doped plasma treated carbon nanofiber as an efficient electrode for symmetric supercapacitor. *J. Energy. Storage.* **2021**, 33, 102150.

[13] Anand, S.; Ahmad, M. W.; Saidi, A. K. A. A.; Yang, D.; Choudhury, A. Polyaniline nanofiber decorated carbon nanofiber hybrid mat for flexible electrochemical supercapacitor. *Mater. Chem. Phys*. **2020**, 254, 123480.

[14] Sun, W.; Gao, G.; Zhang, K.; Liu, Y.; Wu, G. Self-assembled 3D N-CNFs/V_2O_5 aerogels with core/shell nanostructures through vacancies control and seeds growth as an outstanding supercapacitor electrode material. *Carbon*. **2018**, 132, 667–677.

[15] Tang, P.; Han, L.; Zhang, L. Facile synthesis of graphite/PEDOT/MnO_2 composites on commercial supercapacitor separator membranes as flexible and high-performance supercapacitor electrodes. *ACS Appl Mater Interfaces*. **2014**, 6, 10506–10515.

[16] Mansuer, M.; Miao, L.; Qin, Y.; Song, Z.; Zhu, D.; Duan, H.; Lv, Y.; Li, L.; Liu, M.; Gan, L. Trapping precursor-level functionalities in hierarchically porous carbons prepared by a pre-stabilization route for superior supercapacitors. Chin. Chem. Lett. 2022, 10.1016/j.cclet.2022.03.027.

[17] Du, W.; Miao, L.; Song, Z.; Zheng, X.; Lv, Y.; Zhu, D.; Gan, L.; Liu, M. Kinetics-driven design of 3D VN/MXene composite structure for superior zinc storage and charge transfer. J. Power Sources. 2022, 536, 231512.
